# Pregnancy after sexuality preserving cystectomy with urinary diversion for bladder cancer: case report and review of the literature

**DOI:** 10.1186/s12894-022-01076-7

**Published:** 2022-09-05

**Authors:** Flavia Proietti, Leslie Claire Licari, Rocco Simone Flammia, Eugenio Bologna, Veronica Palombi, Emiliano Scarrone, Antonio Tufano, Cosimo De Nunzio, Costantino Leonardo

**Affiliations:** 1 Unit of Urology - Policlinico Umberto I, Department of Maternal-Infant and Urological Sciences, La Sapienza University of Rome, Rome, Italy; 2 Department of Urology – Sant’Andrea Hospital, La Sapienza University of Rome, Rome, Italy

**Keywords:** Bladder cancer, Sex-sparing cystectomy, Robot-assisted surgery, Pregnancy, Case report

## Abstract

**Background:**

Radical cystectomy for bladder cancer represents a high demolitive surgical procedure with a significative impact on quality of life. Sexuality preserving techniques have been proposed in order to improve functional outcomes. Although sex-sparing techniques would provide women with the chance of having pregnancy, experience is still limited when malignant conditions are considered. We report the outcomes of pregnancy and delivery in a 43-year-old woman with a Padua ileal orthotopic neobladder after robot-assisted sexuality preserving cystectomy for muscle-invasive urothelial bladder cancer performed four years earlier, at age 39.

**Case presentation:**

Since pregnancy was confirmed, the patient had been under close urological and gynecological observation. Hydronephrosis and voiding-relating complications were reported and treated by inserting a nephrostomy tubes and indwelling bladder catheter. At the time of delivery, elective caesarian section was performed without complications.

**Conclusions:**

Sexuality preserving cystectomy could be an option in selected and highly motivated young patients with diagnosis of bladder cancer. A multidisciplinary team of experts included gynecologists, urologists, radiologists, anesthesiologists and neonatologists is required for the optimal management of pregnancy and peripartum care in women with urinary diversion.

## Background

Bladder cancer (BC) is the 11th most commonly diagnosed cancer when both genders are considered, with an incidence of ~ 150,000 newly diagnosed cases and 52,000 deaths in Europe in 2012 [1]. Although men are more likely to develop BC than women, the latter present with more advanced disease and have worse survival rates [2]. According to guidelines, radical cystectomy (RC) is the gold standard treatment for muscle invasive bladder cancer (MIBC) cT2-T4 cN0 cM0 and is strongly recommended in non muscle-invasive disease with high risk of progression [3]. Standard RC for women includes removal of the bladder, urethra and adjacent vagina, uterus, distal ureters, and regional lymph nodes [4]. Such a highly demolitive surgical procedure results in a sexual disfunction with a significant impact on health-related quality of life (HR-QoL), especially in young patients [5]. In this scenario, various types of pelvic-organ-preserving techniques, usually named “sex-sparing”, have been proposed in order to optimize sexual and functional outcomes [5]. Although “sex-sparing” technique would provide patients with the chance of having pregnancy, experience is still limited and is related mainly to patients with congenital disorders, neurogenic diseases or trauma. The aim of this study is to provide all the available evidences for pregnancy and childbirth after sexuality preserving cystectomy (SPC) for BC and to report our experience of the first delivery after robot-assisted radical cystectomy (RARC) with orthotopic ileal neobladder reconstruction.

## Case presentation

We report the outcomes of pregnancy in a 43-year old woman with a Padua Ileal Neobladder (VIP, vescica ileale padovana) after SPC, performed after the diagnosis of a malignant tumor at the age of 39. A written consent for case report publication was previously obtained from the patient.

The patient’s history began in 2015 with a diagnosis of muscle invasive bladder cancer for which she underwent robot-assisted SPC with totally intracorporeal neobladder reconstruction, performed by an experienced robotic surgeon (M.G), as previously described [5–7]. Histological examination reported high grade urothelial carcinoma with partial squamous differentiation and concomitant carcinoma in situ (stage pT3b + CIS). A total of 34 lymph nodes were removed and analyzed, resulting with no metastatic involvement (stage pN0). Surgical margins were free from tumor (stage R0). After surgery, the patient was under constant urologic outpatient department surveillance. Left hydronephrosis due to anastomosis fibrotic stenosis was diagnosed 11 months after SPC, and treated with robot-assisted ureteral reimplantation, as previously described [8]. She had continent neobladder requiring clean intermittent catheterization (CIC) once a day before sleeping to avoid nighttime incontinence, with normal renal function.

After 49 months of negative follow-up the patient underwent In Vitro Fertilization (IVF) in the attempt to get pregnant. Since pregnancy was confirmed, the patient had been under close urological and gynecological observation.

At 15th weeks of gestation, flank pain and fever occurred. Blood test showed white blood cell (WBC) count 12.28 × 10^9^/L, C-reactive protein (CRP) 370,600 μg/L, procalcitonin 32.36 ng/mL, serum creatinine 1.26 mg/dL and eGFR 57 mL/min. Ultrasound (US) showed slight upper and left side dislocation of the ONB, causing chronic urinary retention, and high grade left ureterohydronephrosis. A bladder catheter and an US-guided percutaneous nephrostomy tube was placed, with minimal x-ray exposure. *Escherichia coli* has been isolated in urine sample and an intravenous antibiotics infusion was started (meropenem 1gr tid) based on antibiogram. After 10 days of therapy, clinical improvement was achieved, and negative lab tests and urine culture were reported. Patient was discharged without bladder catheter, with the indication to intensify CIC. One month later, right ureterohydronephrosis was diagnosed by US routine assessment. In order to avoid further complicated UTI, an elective US-guided percutaneous nephrostomy tube was placed, with no X-Ray exposure.

During the second and the third trimester of pregnancy, the patient had recurrent asymptomatic bacteriuria, successfully treated at home in accordance with antibiogram reports. Renal function, measured by serum creatinine dosage, remained stable until the conclusion of pregnancy.

**Fig. 1:**
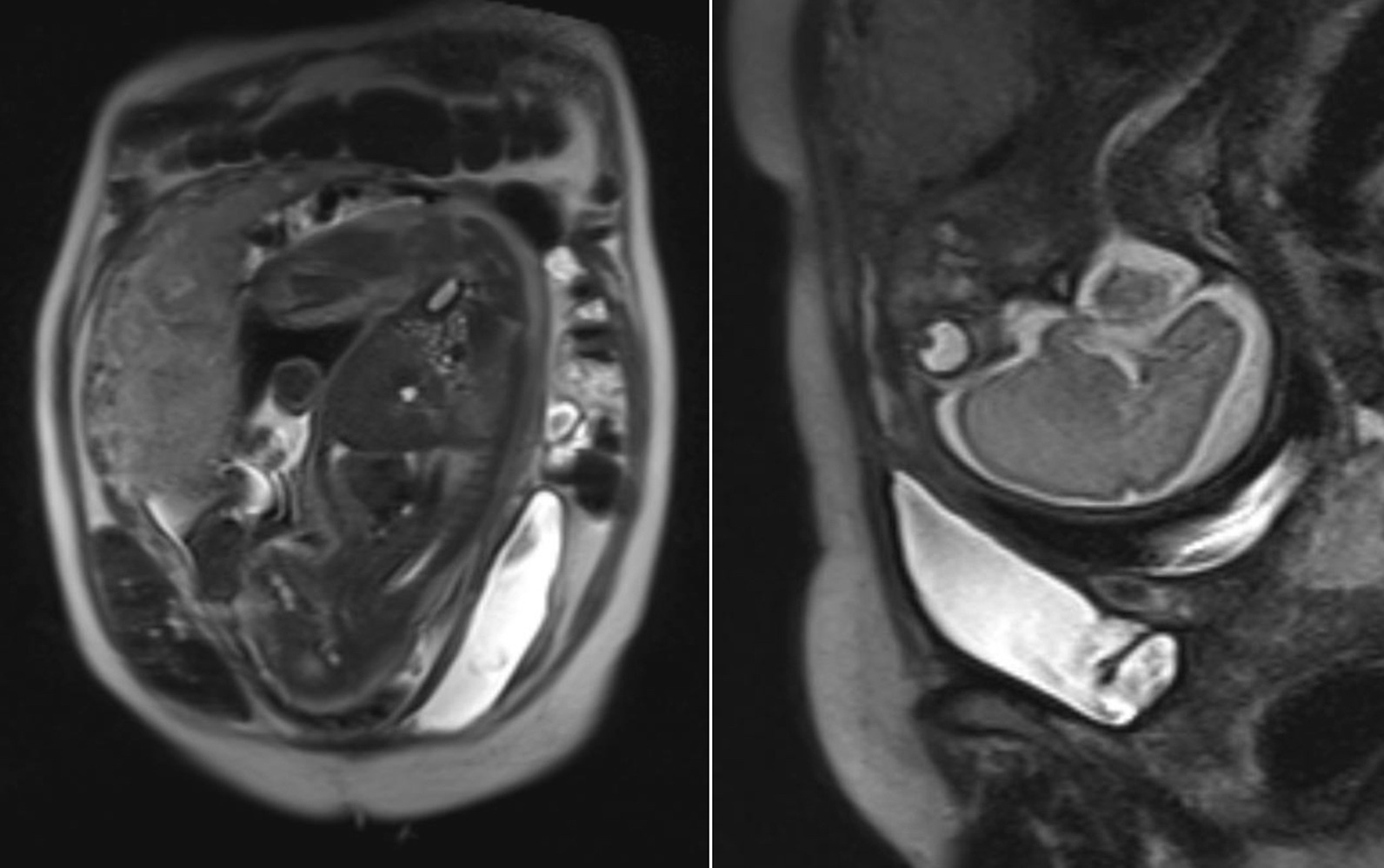
An MRI performed at 31 weeks of gestation shows anatomical relationship and dislocation of neobladder. After retrograde voiding, the neobladder appears to be dislocated in left antero-lateral position to the uterus. No MRI signs of tissue adherence between neobladder and uterus wall are observed

An MRI (Magnetic Resonance Imaging) was performed in December 2019 in order to assess anatomical relationship between uterus and neobladder (Fig. 1).

At 32 weeks of gestation, with the beginning of regular uterine contractions, the decision was to perform an elective cesarean section under continuous epidural anesthesia. The surgery, conducted by two gynecologists and an urologist, lasted 40 min with no perioperative transfusions needed. Neither maternal nor neonatal complications were reported during puerperium observation. Neobladder integrity was checked at the end of the procedure. Patient returned to normal voiding 72 h after delivery and was discharged at postoperative day 5. The nephrostomy tubes were removed 5 weeks later, after bilateral antegrade pyelography showing absence of ureterohydronephrosis, bilaterally. No further complications were reported, and no cancer relapse was diagnosed after 68 months follow-up.

## Discussion and conclusion

In 1922, Knauf firstly described pregnancy after urinary diversion [9]. Since then, the possibility to achieve pregnancy after bladder surgery for benign conditions in patients with urinary diversion, has been extensively reported. In a literature review by Hautmann et al., published in 2007, 252 cases of pregnancy in 188 women after UD were reported [9]. Among this population, authors described only two cases in which RC and UD (Ureterosigmoidostomy and Indiana pouch) for malignant disease had been performed [10, 11] suggesting that, despite the improvement of medical advances, a fertility-sparing approach was a viable option in a selected amount of patients. In the same year, Dhar et al. firstly described the feasibility of SPC with orthotopic diversion in women affected by localized BC, emphasizing functional advantages deriving from this approach [12]. However, pregnancy and delivery in patients with orthotopic neobladder after malignancy was not described until 2011, when Nunnink et al. reported the first case [13], (Table 1).

**Table 1:** Literature review with oncologic and gynecologic findings of pregnancy in women with history of sexuality preserving cystectomy for bladder cancer

Author	Year	Case	Disease histology/stage	Urinary diversion	Age at pregnancy	Years from cancer to pregnancy	Week of delivery	Delivery mode
Barrett [11]	1983	1	Urachal Carcinoma/NA	Ureterosigmoidostomy	NA*	NA*	NA*	Vaginal
Kuczkowski [10]	2004	2	Rabdomyosarcoma/NA	Indiana Pouch	NA*	NA*	37	Cesarean Section
Nunnink [13]	2011	3	Urothelial Cancer/pT3bN0	Indiana Pouch	33	3	40	Vaginal
		4	Urothelial Cancer/pT2N0	Orthotopic Ileal Neobladder (NA)	28	3	NA*	Vaginal
Kolodziej [14]	2016	5	Rabdomyosarcoma/pT2N0	Orthotopic Ileal Neobladder (Studer)	27	13	27	Cesarean Section
Hupe [15]	2016	6	Sarcomatoid Urothelial Cancer/pT3bN0	Orthotopic Ileal Neobladder (Studer)	31	3	37	Cesarean Section
Our case	2020	7	Urothelial Cancer/pT3bN0	Orthotopic Ileal Neobladder (VIP)	43	4	32	Cesarean Section

*NA not available

Regarding surgical approach, open surgery was performed at the time of cystectomy in all cases described; to our knowledge, our experience represents the first one in which minimally invasive SPC (robot-assisted) was performed.

When histopathological findings after radical cystectomy are considered, urachal carcinoma, rabdomyosarcoma and urothelial cancer have been reported [10, 11, 13], with T-stage T2-T3b and absence of lymph node involvement [13, 14] in all cases.

During pregnancies after urinary diversion, displacement of neobladder (laterally to the right or left side) was observed [14], due to growing uterus. Most common conditions described are related mainly to neobladder emptying and ureteral compression, events that usually occurred during second trimester [14]. Management of hypercontinence usually consisted in starting or intensifying CIC. Sometimes, a placement of indwelling bladder catheter is required. Ureterohydronephrosis due to ureteral compression and recurrent urinary tract infections are the most frequent complications reported [13, 14]. Scheduled genitourinary US and urinalysis (at least once a month) were suggested [14], although management of asymptomatic bacteriuria was not assessed. When secondary pyelonephritis was suspected, intravenous antibiotics infusion and nephrostomy tube insertion have been performed [13, 14].

Bowel movement abnormalities due to adhesions and altered anatomy and metabolic complications are also reported [9].

Regarding delivery, both vaginal and caesarian section have been reported. In our patient, similar to Kolodizej experience, caesarian section was performed with incision in higher uterine segment in order to reduce the risk of neobladder or ureteral injury. The possibility of temporarily interrupting the flow of the internal iliac arteries with balloon catheters has also been described [14].

It has been reported necessity to continue CIC and/or to maintain bladder catheter and nephrostomy tube until uterus involution. We opted to perform antegrade pyelography, 5 weeks after delivery, before proceeding to nephrostomy tubes removal.

With the spread of sexuality preserving techniques and minimally-invasive surgery, the number of pregnancies and deliveries in patients with history of radical cystectomy and urinary diversion is expected to increase. Despite the number of possible complications, management of this particular setting of patients, that require close surveillance, is possible by relying on a multidisciplinary team, including urologists, gynecologists and neonatologists.

## Data Availability

All data generated and analysed during this study are included in this published article.

## Abbreviations

BC: Bladder cancer
CIC: Clean intermittent catheterization
HR-QoL: Health-related quality of life
IVF: In vitro fertilization
MIBC: Muscle-invasive bladder cancer
MRI: Magnetic resonance imaging
RARC: Robot-assisted radical cystectomy
RC: Radical cystectomy
SPC: Sexuality preserving cystectomy
US: Ultrasound

## References

[1] Ferlay J, Steliarova-Foucher E, Lortet-Tieulent J, Rosso S, Coebergh JWW, Comber H (2013). Cancer incidence and mortality patterns in Europe: estimates for 40 countries in 2012. Eur J Cancer.

[2] Mungan NA, Aben KKH, Schoenberg MP, Visser O, Coebergh JWW, Witjes JA (2000). Gender differences in stage-adjusted bladder cancer survival. Urology.

[3] Witjes JA, Bruins HM, Cathomas R, Compérat EM, Cowan NC, Gakis G (2020). European association of urology guidelines on muscle-invasive and metastatic bladder cancer: summary of the 2020 guidelines. Eur Urol.

[4] Stenzl A, Nagele U, Kuczyk M, Sievert KD, Anastasiadis A, Seibold J (2005). Cystectomy: technical considerations in male and female patients. EAU Updat Ser.

[5] Tuderti G, Mastroianni R, Flammia S, Ferriero M, Leonardo C, Anceschi U (2020). Sex-sparing robot-assisted radical cystectomy with intracorporeal Padua Ileal Neobladder in female: surgical technique, perioperative, oncologic and functional outcomes. J Clin Med.

[6] Simone G, Papalia R, Misuraca L, Tuderti G, Minisola F, Ferriero M (2018). Robotic intracorporeal Padua Ileal Bladder: surgical technique, perioperative oncologic and functional outcomes. Eur Urol.

[7] Brassetti A, Cacciamani G, Anceschi U, Ferriero M, Tuderti G, Miranda G (2020). Long-term oncologic outcomes of robot-assisted radical cystectomy (RARC) with totally intracorporeal urinary diversion (ICUD): a multi-center study. World J Urol.

[8] Tuderti G, Brassetti A, Minisola F, Anceschi U, Ferriero M, Leonardo C (2019). Transnephrostomic indocyanine green-guided robotic ureteral reimplantation for benign ureteroileal strictures after robotic cystectomy and intracorporeal neobladder: step-by-step surgical technique, perioperative and functional outcomes. J Endourol.

[9] Hautmann RE, Volkmer BG (2007). Pregnancy and urinary diversion. Urol Clin North Am.

[10] Kuczkowski KM, Hay B (2004). Peripartum care of the parturient with Indiana continent urinary diversion: a need for a multidisciplinary approach. Ann Fr Anesth Reanim.

[11] Barrett RJ, Peters WA (1983). Pregnancy following urinary diversion. Obstet Gynecol.

[12] Dhar NB, Kessler TM, Mills RD, Burkhard F, Studer UE (2007). Nerve-sparing radical cystectomy and orthotopic bladder replacement in female patients. Eur Urol.

[13] Nunnink CJ, de Vries RR, Meinhardt W, van der Poel HG, Bex A, Horenblas S (2011). Zwangerschap na seksualiteit preserverende cystectomie wegens blaaskanker [Pregnancy following sexuality-preserving cystectomy for bladder carcinoma]. Ned Tijdschr Geneeskd.

[14] Kołodziej A, Krajewski W, Tupikowski K, Małkiewicz B, Dembowski J, Zimmer M (2016). Pregnancy and delivery in a patient with a Studer orthotopic ileal neobladder. Cent Eur J Urol.

[15] Hupe MC, Merseburger AS, Günter HH, Wüstemann M, von Kaisenberg CS (2016). Successful pregnancy and neobladder subsequent to muscle invasive bladder cancer and fertility preserving surgery: case report and review of the literature. Urol Int.

